# Ambivalent Stereotypes and Persuasion: Attitudinal Effects of Warmth vs. Competence Ascribed to Message Sources

**DOI:** 10.3389/fpsyg.2021.782480

**Published:** 2022-01-27

**Authors:** Roman Linne, Melanie Schäfer, Gerd Bohner

**Affiliations:** ^1^Social Psychology, Helmut Schmidt University, Hamburg, Germany; ^2^Department of Psychology, Bielefeld University, Bielefeld, Germany

**Keywords:** persuasion, stereotype content model, source effects, motives, warmth, competence

## Abstract

The stereotype content model (Fiske et al., 2002) defines warmth and competence as basic dimensions of social judgment, with warmth often dominating perceptions; it also states that many group-related stereotypes are ambivalent, featuring high levels on one dimension and low levels on the other. Persuasion theories feature both direct and indirect source effects (Bohner et al., 1995). Combining both the approaches, we studied the persuasiveness of ambivalently stereotyped sources. Participants (total *n* = 296) read persuasive arguments attributed to groups stereotyped as either low in competence but high in warmth (e.g., housewives) or vice versa (e.g., lawyers). In Study 1, high competence/low warmth sources were more persuasive than low competence/high warmth sources. In Study 2, this pattern replicated when an accuracy motive had been induced, whereas it reversed when a connectedness motive had been induced. These source effects were direct, that is, independent of message processing. We discuss our findings in terms of the persuasiveness of warmth vs. competence of the source as being dependent on recipient motivation; we also consider theoretical implications and perspectives for future research.

## Introduction

Groups are both targets of social perception (Cuddy et al., 2008) and sources of social influence (Moscovici, 1976). In this article, we combine these two aspects and examine how the way in which groups are stereotyped may affect the persuasive impact of these groups. We focus on ambivalent group stereotypes in terms of the stereotype content model (SCM; Fiske et al., 2002), comparing groups perceived to be high in competence but low in warmth (e.g., lawyers) with groups perceived to be high in warmth but low in competence (e.g., disabled people) as sources of persuasion. We show that, by default, high competence/low warmth groups may have a persuasive advantage over high warmth/low competence groups (Study 1), but also that this advantage may reverse if recipients are connectedness-motivated (Study 2). In order to elaborate our research questions, we address theorizing on source characteristics and processing motives in persuasion, as well as the role of ambivalent source cues both generally and in relation to the SCM.

### Source Characteristics in Persuasion

The study of source characteristics has a long history in persuasion research and theorizing. As early as in the 4th century BCE, Aristotle discussed using the authority and credibility of the speaker as a skillful means of persuasion (Krapinger, 1999). In modern social psychology, source factors were prominently addressed by the approach of the Yale group to persuasion. Among the specific attributes studied were source expertise and trustworthiness (Hovland and Weiss, 1952). Effects of the message source are also prominent in more recent processing models of persuasion. In both the elaboration likelihood model (ELM; Petty and Cacioppo, 1986) and the heuristic-systematic model (HSM; Chaiken et al., 1989), source attributes, such as likability, expertise, attractiveness, and majority status have been conceptualized as cues to message validity. As such, source attributes may influence attitude judgments in at least three ways.

Firstly, source attributes often exert a direct main effect on attitudes, especially when recipients are unwilling or unable to invest much effort in message processing. Under such conditions, recipients may follow simple heuristics, such as “experts’ statements are valid” or “I usually agree with people I like,” and thus agree more with expert or likable sources than with nonexpert or dislikable sources, respectively (Bohner et al., 1995). Secondly, when recipients are motivated and able to process extensively, source cues may bias the processing of message arguments and may thus exert a more indirect effect on attitudes (Bohner et al., 1995; Petty and Wegener, 1999). In particular, when message arguments are ambiguous or open to interpretation, a positive (negative) cue may make a given argument appear more (less) convincing (Chaiken and Maheswaran, 1994; Erb et al., 1998). Thirdly, source information may be a factor that motivates more or less extensive processing of a message. More extensive processing may result, for example, when source attributes are surprising in a given context (e.g., when a counterattitudinal position or a patently weak product is supported by a majority source Maheswaran and Chaiken, 1991; Baker and Petty, 1994). A similar threefold effect of source information may also be derived from the unimodel of persuasion (Kruglanski and Thompson, 1999; Erb et al., 2003; for discussion, see Bohner et al., 2008; Bohner and Dickel, 2011).

### Source Cues and Processing Motives

Persuasion theorists have often treated the effects of different source attributes as functionally interchangeable (Petty and Cacioppo, 1986; Chaiken et al., 1989; but see, e.g., Shavitt et al., 1994). According to this view, what matters for persuasion is whether a source attribute is positive (e.g., high expertise or likability) or negative (e.g., low expertise or likability), but not which particular attribute (expertise vs. likability) is presented. Another research tradition, however, suggests that specific source characteristics may interact with specific motivational circumstances. In the words of Jones and Thibaut (1958), “If we can successfully identify the goals for which an actor is striving in the interaction situation, we can begin to say something about the cues to which he will attend, and the meaning he is most likely to assign them” (p. 152). Some persuasion studies have shown that the motivation of the recipients may indeed moderate the influence of source factors. For example, Imhoff and Erb (2009) showed that recipients who were higher in the need for uniqueness (either chronically or as the result of an experimental manipulation) were more influenced by minority (vs. majority) sources. Indeed, many studies have shown effects of personalized matching in persuasion (for a review, see Teeny et al., 2021). Framing aspects of the persuasion process (source, message, etc.) in terms that fit the recipient generally (but not always) increases the likelihood of successful persuasion. For example, participants concerned with their social image are especially susceptible to messages highlighting the social benefits of a given attitude object (Lavine and Snyder, 1996).

Of particular importance to research in social cognition are the motives of mastery and connectedness. Striving for mastery means people seek to understand their social environment, guided by accurate and unbiased information, whereas striving for connectedness means people seek support and acceptance from others (Smith et al., 2015, p. 17). The mastery motive has been prominent in persuasion research under the label of “accuracy motivation” (Chaiken and Stangor, 1987). Individuals following an accuracy motive strive for an open-minded representation of the state of the world, including appropriate and unbiased attitudes (Bohner et al., 1995, 2008; Chen and Chaiken, 1999; Wood, 2000). Thus, an accuracy motive may help recipients focus on cues that inform them about the correctness of a persuasive message, such as source expertise (Keller et al., 2000).

In contrast, the connectedness motive, often dubbed as “impression motivation” within a persuasion framework (Bohner et al., 1995, 2008; Chen and Chaiken, 1999), may help recipients focus on other types of information, which are linked to the theme of social interaction. Individuals following an impression motive strive to obtain acceptance from others and to maintain smooth and rewarding interpersonal relations. In a persuasion setting, impression-motivated recipients may thus prefer “getting along” over “getting at the truth” (Chen et al., 1996) and may rely on cues that signal social harmony and warmth (Bohner et al., 1995, 1998).

### Warmth and Competence as Fundamental Dimensions of Social Judgment

Stereotypes may be seen as cognitive instruments that provide important information about outgroups. In the SCM, Fiske et al. (2002) proposed the concepts of warmth and competence as two fundamental dimensions that people use to judge social groups (for related conceptual dichotomies, see Bakan, 1966; Rosenberg et al., 1968; Abele and Wojciszke, 2007, 2014). Groups may thus be judged on a warmth dimension, including attributes such as cold, warm, hostile, good-natured, or dishonest, and on a competence dimension, including attributes such as incompetent, capable, dull, intelligent, or inefficient (Fiske et al., 2002). The two dimensions may serve as cues indicating whether others (a) have good or bad intentions (warmth) and (b) are able to act on those intentions (competence). While some group stereotypes are only positive (or only negative) on both dimensions, an important insight of the SCM is that many social groups are evaluated positively on one dimension and negatively on the other. Examples for such ambivalent stereotyping would be the groups of housewives (perceived to be high on warmth and low on competence) or lawyers (perceived to be high on competence but low on warmth).

Cuddy et al. (2008) argued that the warmth dimension is of greater social and cognitive significance than the competence dimension (Abele and Wojciszke, 2014). Warmth has been shown to be more relevant for social interaction (Baumeister and Leary, 1995; Wojciszke et al., 1998), which in turn is indispensable for human existence. Also, it may be adaptive to focus on the warmth of others because recognizing good or ill intent in others may bear directly on the chances of survival of the perceiver. Accordingly, research has shown that warmth-related trait words are both recognized faster and categorized faster in terms of their valence than competence-related trait words (Ybarra et al., 2001; Abele and Bruckmüller, 2011; de Lemus et al., 2013). There is also evidence for a primacy of warmth in person perception. When participants were asked to write down the most important traits, eight of the 10 most mentioned words referred to warmth (e.g., sincere, honest, loyal, and reliable), whereas only two (e.g., intelligent) referred to competence (Wojciszke et al., 1998).

However, other studies indicate that the primacy of warmth vs. competence may depend on the task context. For example, participants chose warmth-related traits to judge the trustworthiness of others, but chose competence-related traits to judge the negotiation skills of others (Wojciszke et al., 1998). Furthermore, Abele and Wojciszke (2007) showed that participants rated agency-related traits (which also convey competence) as more important for themselves, but rated communion-related traits (which convey warmth) as more important for their evaluation of others. In research on persuasion, Dubois et al. (2016) have proposed a power-matching effect between communicators and audience: High-power sources may generate messages emphasizing competence information, which may be more effective in persuading high-power audiences, whereas low-power sources may emphasize warmth information, which may be more effective in persuading low-power audiences.

To sum up, while there is evidence for matching effects in general and context-dependency in the relative importance of warmth vs. competence specifically, it seems that there might be a primacy of warmth at least in an interpersonal context.

### Warmth and Competence in Persuasion: The Present Research

There are many studies showing the persuasive effects of *individual* source attributes that are similar to the SCM dimensions. Characteristics such as expertise (Petty et al., 1981) or likability (Chaiken, 1980; Ziegler and Diehl, 2001; Reinhard and Messner, 2009) have been shown to increase the influence of a source. However, it is more difficult to predict the influence of *ambivalent combinations* of source characteristics (e.g., a likable layperson vs. a dislikable expert). Because such combinations may be surprising, they may cause more systematic processing of message content (Ziegler and Diehl, 2001; Ziegler et al., 2002).

But what about ambivalently stereotyped *groups* as agents of persuasion? Research on the SCM (Fiske et al., 2002; Cuddy et al., 2008) suggests that there are many groups being stereotyped as competent but cold (e.g., rich people, career women, and lawyers) and many groups being stereotyped as warm but incompetent (e.g., people with disabilities, housewives, and elderly people). Different from the study by Ziegler et al. (2002), encountering such groups as sources of persuasion would probably *not* appear surprising or unexpected, because the opposing valences on the warmth and competence dimensions are part of a holistic perception of the group. Nonetheless, as Cuddy et al. (2008) argued, just mentioning a stereotyped group may evoke certain feelings, thoughts, and behaviors. The approach to study the effects of ambivalently stereotyped groups as sources of persuasion may yield new insights into the dynamics of perceived warmth and competence. Therefore, for the first time, the present work combines theoretical approaches from SCM and persuasion research.

While the source cues of expertise and likability seem close to the concepts of competence and warmth as defined in the SCM, these pairs of concepts are not interchangeable. The concept of competence is broader than the attribute of expertise often used in persuasion experiments. Whereas many experiments have used a specific expertise cue (e.g., a “Professor of Ecology and Infrastructure” arguing in favor of a traffic project; Bohner et al., 2002), the concept of competence encompasses many different traits but not necessarily any specific knowledge about a specific topic. Similarly, the concept of warmth may encompass the attribute of likability, but also a variety of other traits.

The use of ambivalently stereotyped groups as persuasion sources opens up the possibility of measuring the effects of both warmth and competence simultaneously. Thus, results may shed light on the nature of the relative or shared influence of the basic dimensions; for example, additive effects of warmth and competence, potential compensatory effects, or effects where one dimension prevails (such as a general primacy of warmth that may extend to the persuasion context). In two studies, we presented, as sources of persuasion, social groups that were perceived either as high on competence but low on warmth (hc/lw) or as low on competence but high on warmth (lc/hw). In addition, we used students as the source in a third condition. The rationale was that students would represent a group supposedly high on both dimensions due to being an ingroup for participants (Cuddy et al., 2008). Thus, the inclusion of students (hc/hw) allowed for the testing of potential additive effects of warmth and competence. In both studies, differently stereotyped groups presented arguments for a shower foam, a product of daily use with an innovative touch, for which neither warmth nor competence conveyed any specific expertise. Several aspects of the shower foam were advertised with arguments that varied in strength (see Supplementary Material), and their evaluation was assessed separately; thus, argument strength was varied as a within-subjects factor (Erb et al., 2005).

In the light of previous findings suggesting a primacy of warmth (as discussed above), it may seem plausible also in the case of ambivalently stereotyped groups as message sources that warmth will have more persuasive impact than competence. However, there is also ample research showing that the relative importance of each dimension may depend on the situation. Therefore, if message recipients are more accuracy-motivated, there might be a situational primacy of competence. Thus, we entertained two competing hypotheses: Either there is a general primacy of warmth that extends to persuasion, which means that groups stereotyped as being lc/hw should be more influential than groups stereotyped as hc/lw. Alternatively, which type of group is more influential may depend on a match between its perceived attributes and recipients’ motivation; this alternative was more specifically examined in Study 2.

When we say that groups are “influential,” as discussed above, this may involve at least three types of processes: (a) a direct effect of source attributes on attitudes, (b) an indirect effect of source attributes on attitudes *via* biasing the favorability of message-related thoughts, or (c) an effect of source attributes on the extent of message processing, which would imply an interaction effect of source attributes and argument quality. Our study designs allowed for any of these potential effects to be detected, by including source manipulations, assessments of message-related thoughts and their favorability, and variations of argument quality.

## Study 1

### Method

#### Participants and Design

A total of 92 participants (45 women, 47 men; *M*_*age*_ = 23.88, *SD*_*age*_ = 3.83) completed the study and consented to their data being used. Most participants (*n* = 82) reported to be university students. Participants were randomly allocated to one of five source group conditions (for group selection, see Pilot Study 1, Supplementary Material): housewives (*n* = 17), career women (*n* = 18), lawyers (*n* = 17), disabled people (*n* = 16), or students (*n* = 24). With this sample size, the statistical power (1 — beta) for detecting medium-sized (*f* = 0.25) contrast effects at an alpha level of 0.05 within an ANOVA was 0.66 (Faul et al., 2007).

#### Procedure

Participants took part in an online survey that contained a cover story, a persuasive message (a product review), and a questionnaire. They learned that their task was to evaluate the concept of a new website featuring product reviews. The website was said to use the innovative approach of sorting product reviews by testers belonging to different social groups, so that customers could access the reviews of social groups whose opinions they considered relevant. Supposedly in order to illustrate the concept, participants were asked to read an exemplary product review about a shower foam that was allegedly based on the comments of 153 lawyers (or career women, housewives, disabled people, students, respectively). After reading the review, participants indicated their attitudes toward the shower foam. Further, they were asked to write down any thoughts that came to mind while reading the arguments, and then to rate each specific aspect of the shower foam for which an argument had been presented (e.g., its eco-friendliness). In order to maintain the cover story, participants were then asked to rate the website and propose a name for it. The questionnaire ended with ratings of the source groups, perceived similarity, and an open-ended suspicion check; then participants were debriefed and asked once more for their consent to their data being used.

#### Perceived Similarity

Participants were asked to indicate their perceived similarity to each of the five source groups on a 7-point scale ranging from 1 = *not at all similar* to 7 = *extremely similar*.

#### Persuasive Message and Argument Strength

The product review featured seven arguments that had been selected in Pilot Study 2 (see Supplementary Material). As these arguments varied in strength (strong, moderate, and weak) and participants separately rated each aspect that was addressed by a given argument, an individual systematic-processing index could be calculated by taking the difference in participants’ evaluation of strong vs. weak aspects (see Erb et al., 2005).

#### Attitude Toward Product

Two items addressed the participants’ attitude toward the shower foam: “How likely is it that you would buy this product for yourself?” and “How likely is it that you would recommend this product to a friend?” Both were followed by a 7-point scale ranging from (1 = *not at all* to 7 = *very*). These were averaged to form an index of the overall attitude, *r*(90) = 0.81. (A third item, addressing the price that participants would be willing to pay for the shower foam, would have considerably decreased the reliability and was thus not included.)^1^

#### Thought Favorability

Participants were provided with ten boxes and instructed to list one thought per box, using as few or many of the boxes as they required. Each thought that participants listed was later categorized by two independent coders as favorable, neutral, or unfavorable toward the shower foam. Then, for each coder, an index of thought favorability was computed by subtracting the number of unfavorable thoughts from the number of favorable thoughts and dividing the result by the total number of thoughts. As the inter-coder reliability for this index was very high (Spearman-Brown coefficient = 0.922), the final index of thought favorability was computed by averaging across the two coders. Three participants who had not listed any thoughts received a thought favorability index of zero.

#### Attitudes Toward Strong and Weak Aspects

Participants’ ratings of the two aspects supported by strong arguments [cleansing and care; *r*(90) = 0.66] and the two aspects supported by weak arguments [price and handling; *r*(90) = 0.44], respectively, were averaged into separate indices.

#### Source Ratings

At the end of the questionnaire, participants were asked to rate their own source group, as well as each of the other four source groups used in the experiment, on the dimensions of warmth (items: *likable*, *warm-hearted*, *good-natured*, and *friendly*) and competence (items: *competitive*, *competent*, *independent*, and *intelligent*). The response scale went from 1 = *not at all* to 7 = *completely*.

## Results

### Effectiveness of Source Manipulation

An ANOVA on perceived warmth confirmed the effectiveness of the source manipulation, *F*(2, 89) = 27.82, *p* < 0.001, η^2^ = 0.385, 95% CI (0.222, 0.502). As intended, the lc/hw groups (housewives and disabled people) were perceived to be higher in warmth (combined *M* = 5.03, *SD* = 1.03) than the hc/lw groups (career women and lawyers) (*M* = 3.67, *SD* = 0.83), *t*(89) = 6.18, *p* < 0.001, *d* = 1.50, 95% CI (0.97, 2.02). The hc/hw group (students) was also rated higher in warmth (*M* = 5.23, *SD* = 0.83) than career women and lawyers, *t*(89) = 6.49, *p* < 0.001, *d* = 1.72, 95% CI (1.12, 2.29) but did not differ from housewives and disabled people, *t* < 1.

An ANOVA on perceived competence also confirmed the effectiveness of the source manipulation, *F*(2, 89) = 12.78, *p* < 0.001, η^2^ = 0.223, 95% CI (0.079, 0.351). As intended, the hc/lw groups (career women and lawyers) were perceived to be higher in competence (*M* = 5.61, *SD* = 0.92) than the lc/hw groups (housewives and disabled people) (*M* = 4.23, *SD* = 1.29), *t*(89) = 5.05, *p* < 0.001, *d* = 1.22, 95% CI (0.71, 1.73). The hc/hw group (students) (*M* = 5.03, *SD* = 1.19) was perceived to be more competent than the lc/hw groups, *t*(89) = 2.65, *p* = 0.010, *d* = 0.71, 95% CI (0.17, 1.24), but also somewhat less competent than the hc/lw groups; *t*(89) = −1.94, *p* = −0.055, *d* = −0.52, 95% CI (0.12, −1.04).

Furthermore, as expected, participants reported higher perceived similarity to their ingroup, students (*M* = 5.14, *SD* = 1.39), than to any of the other source groups (combined *M* = 2.84, *SD* = 1.68), *t*(89) = 6.23, *p* < 0.001, *d* = 2.96, 95% CI (1.93, 3.98).

### Product Attitude

In order to examine source effects on participants’ attitude toward the shower foam, we computed a one-way ANOVA with three levels of the source factor (lc/hw vs. hc/lw vs. hc/hw) including *a-priori* contrasts. The ANOVA yielded a significant overall effect of the source, *F*(2, 89) = 5.27, *p* = 0.007, η^2^ = 0.106, 95% CI (0.009, 0.223). Contrast analyses showed that attitudes toward the product were significantly more positive when it was recommended by the hc/lw groups (*M* = 3.49, *SD* = 1.30) than by the lc/hw groups (*M* = 2.45, *SD* = 1.42), *t*(89) = 3.01, *p* = 0.003, *d* = 0.73, 95% CI (0.24, 1.22). Attitudes were also more positive when the product was recommended by students (*M* = 3.40, *SD* = 1.57) than by the lc/hw groups, *t*(89) = 2.48, *p* = 0.015, *d* = 0.67, 95% CI (0.13, 1.20). The student condition and the hc/lw group conditions did not differ, *t* < 1. These results suggest that high competence, rather than high warmth, of a message source is the relevant factor in changing attitudes.

### Thought Favorability

A similar ANOVA on thought favorability showed a trend toward group differences, *F*(2,89) = 2.64, *p* = 0.077, η^2^ = 0.056, 95% CI (0, 0.156). However, thought favorability did not differ between hc/lw (*M* = −0.17, *SD* = 0.38) and lc/hw conditions (*M* = −0.26, *SD* = 0.58), *t* < 1. Thoughts were more favorable toward the shower foam when students (*M* = +0.05, *SD* = 0.52) rather than lc/hw groups were the source, *t*(89) = 2.27, *p* = 0.026, *d* = 0.61, 95% CI (0.07, 1.14). There was no difference in thought favorability between students and hc/lw groups, *t*(89) = 1.64, *p* = 0.105. Thus, the favorability of thoughts about the product was not much affected by competence or warmth of the source groups. We did, however, observe a high correlation of thought favorability and product attitudes, *r*(90) = 0.66, *p* < 0.001, 95% CI (0.53, 0.76). This suggests that participants were generally engaged in the systematic processing of the message, independent of the source. When thought favorability was included as a covariate in an ANCOVA on product attitude with the three levels of the source factor (lc/hw vs. hc/lw vs. hc/hw) as a between-subjects factor, the results of the initial ANOVA replicated. Thought favorability was significant as a covariate, *F*(1, 88) = 70.59, *p* < 0.001, η^2^ = 0.445, 95% CI (0.290, 0.560), but its inclusion did not alter the main effect of the source, *F*(2, 88) = 5.72, *p* = 0.005, η^2^ = 0.115, 95% CI (0.012, 0.234).

### Attitudes Toward Strong and Weak Aspects

As a further test of systematic processing, we examined participants’ attitudes toward strong and weak aspects of the message in a mixed-model 2 × 3 ANOVA, with aspects (strong vs. weak) as a within-subjects factor and source groups (lc/hw vs. hc/lw vs. hc/hw) as a between-subjects factor. Aspects that were promoted with strong arguments (*M* = 6.27, *SD* = 1.73) were evaluated much more positively than aspects promoted with weak arguments (*M* = 3.96, *SD* = 2.06), *F*(1,89) = 83.92, *p* < 0.001, η^2^ = 0.485, 95% CI (0.335, 0.593), which indicates an overall systematic processing. In addition, aspects of the product were generally evaluated more positively when the sources were hc/lw groups (*M* = 5.61, *SD* = 1.49) rather than hw/lc groups (*M* = 4.52, *SD* = 1.51), with the hc/hw source (students) condition falling in between (*M* = 5.21, *SD* = 1.22), *F*(2,89) = 5.1, *p* = 0.008, η^2^ = 0.103, 95% CI (0.008, 0.219). A *post-hoc* test (Duncan) comparing the levels of the source factor revealed that the hc/lw and lc/hw conditions were significantly different from each other (*p* < 0.01), whereas the hc/hw (student) condition did not differ from either (*p* > 0.06). Importantly, there was no significant interaction of argument quality and source groups, *F*(2,89) = 1.80, *p* > 0.17. These findings suggest that participants were generally processing systematically, and there was an independent effect of the source group, with competence rather than warmth being the decisive feature. The absence of an interaction effect suggests that source cues influenced attitudes directly and did not affect the extent of message processing.

### Perceived Warmth, Competence, and Thought Favorability as Concurrent Predictors of Product Attitude

As a final test of the role of competence vs. warmth in the influence of source groups on attitudes, we conducted a multiple regression analysis with participants’ ratings on perceived competence and perceived warmth of their source group, as well as thought favorability, as concurrent predictors of the overall attitude. This analysis revealed that thought favorability was the strongest predictor of attitude, beta = 0.65, 95% CI (0.50, 0.81), *t*(88) = 8.35, *p* < 0.001. Perceived competence was also a positive predictor of attitude, beta = 0.18, 95% CI (0.03, 0.34), *t*(88) = 2.31, *p* = 0.023, whereas warmth was a *negative* predictor, beta = −0.16, 95% CI (−0.32, −0.01), *t*(88) = −2.09, *p* = 0.039. These results further corroborate a pattern where perceptions of high competence combined with low warmth of a message source impact attitudes directly, independent of the systematic processing of message content.

## Discussion

We had proposed a primacy of warmth (Cuddy et al., 2008) as one plausible mechanism behind persuasion by ambivalently stereotyped groups. Instead, we found the opposite; groups stereotyped as being high in competence but low in warmth were more successful in promoting the shower foam. Also, there was no additive effect of warmth and competence. Students, as an ingroup perceived as being high on both dimensions, were as influential as the groups that were high only on competence. They were, however, not more influential, despite being perceived as warmer. Indeed, regression analysis showed higher degrees of warmth attributed to the source being negatively related with the product evaluation. Furthermore, rather than groups attributed with having the highest specific expertise (housewives; see Pilot Study 3, Supplementary Material), it was groups attributed with having the highest general competence that were the most influential. We also proposed the possibility of indirect source effects *via* biased processing. However, results indicated that there was only a direct effect of the competence dimension. While thought favorability correlated with the general evaluation of the shower foam, this correlation was independent of the source. Furthermore, if the source had served as a cue motivating a greater extent of message elaboration, we would have expected different degrees of systematic processing depending on the source. Instead, we found no interaction of source and argument strength but additive main effects of each.

Besides a general primacy of warmth, we had also considered the possibility that either one dimension or the other might be more influential depending on the context. In Study 1, no specific manipulation of context was used. However, it is conceivable that being in an experimental situation itself created a concern for accuracy in participants (see Hilton and Darley, 1991, p. 259). There was no personal interaction (Wojciszke et al., 1998; Abele and Wojciszke, 2007), and participants were asked to complete an evaluation task. Thus, the experimental situation might have activated an accuracy motive rather than an impression motive. To address potential context effects more directly in Study 2, we examined if persuasion effects of ambivalently stereotyped sources reflect an interaction of the source and a manipulation of the current processing motive.

## Study 2

As discussed, the relative influence of the warmth and competence dimensions may be both context-dependent and motive-dependent (Wojciszke et al., 1998). Study 1 showed a higher influence of groups associated with high competence rather than high warmth. An accuracy motive, as a potential default in the experimental situation, was discussed as a possible explanation. If the effects found in Study 1 can be explained *via* a source × (default) motivation interaction, activation of a goal that cannot be satisfied by accuracy (but rather by connectedness) might result in different effects. Therefore, in Study 2, we experimentally induced either a connectedness motive or an accuracy motive. In the connectedness conditions, we expected to see a stronger influence of warmth-related cues (i.e., sources high in warmth). In the accuracy conditions, in contrast, we expected to see a stronger influence of competence-related cues (i.e., sources high in competence).

In Study 1, we had only found direct effects of source competence on attitudes, but found no effects of the source on either thought favorability or message elaboration. In order to test whether these results would replicate in Study 2, we used a similar design as in Study 1, with a persuasive message about the shower foam, a within-subjects variation of argument strength, and a thought-listing task. We hypothesized that the hc/lw (vs. lc/hw) sources would have a stronger influence when an accuracy motive would be induced, whereas the lc/hw (vs. hc/lw) sources would have a stronger influence when a connectedness motive would be induced. Finally, we predicted the hc/hw source (students) to be successful in promoting the shower foam in both motive conditions. However, as no additive effect of warmth and competence was expected, we did not predict them to be more successful than the matching ambivalently stereotyped groups (e.g., hc/lw groups when accuracy is primed). Results of Study 1 had indicated only direct source effects rather than biasing effects or effects *via* the extent of processing. Nevertheless, all three types of influence were again examined.

### Method

#### Participants and Design

A total of 203 participants (133 women, 70 men; *M*_*age*_ = 22.33, *SD*_*age*_ = 3.99) completed the study and consented to their data being used. Most participants (*n* = 195) reported to be university students. Participants were randomly allocated to one of the conditions of a 5 (source group: housewives, disabled people, career women, lawyers, students) × 2 (motive priming: accuracy, connectedness) between-subjects design. With this sample size, the statistical power (1—beta) for detecting medium-sized contrast effects (*f* = 0.25) within an ANOVA design was found to be 0.94 (Faul et al., 2007).

#### Procedure and Motive Priming

Study 2 was conducted on desktop computers in a laboratory. Participants learned that the experiment was about the relationship between imagination and text comprehension. Afterward, they were subjected to a motive-priming task adapted from Bortz (2013). They were told to imagine being in a forest, playing a game called “the net,” which features a parcours of ropes stretched between trees that the players have to navigate through. Participants in the accuracy conditions were instructed to imagine being very careful not to make any mistakes and not to touch any of the ropes; participants in the connectedness conditions were instructed to imagine cooperating with other players and holding their hands while performing the task, with touching the ropes not being a problem. After reading the instructions, all participants were shown a picture of “the net” (a black and white picture of a rope stretched between trees), asked to wear headphones to silence outside noises, and to intensively imagine the task for 1 min. The priming task ended with a signal tone and was followed by a slightly modified version of the questionnaire already used in Study 1 (see section below for detail). After completing the questionnaire, participants were thanked, debriefed, and asked once more for consent.

#### Motive Manipulation Check

Seven items were used to assess the effectiveness of the motive priming: Three items addressed a connectedness motive (e.g., “*In your imagination, did you work as a team when playing the game?*”; 1 = *not at all* to 7 = *very much*), three items addressed an accuracy motive (e.g., “*In your imagination, was it important to complete the game without touching the ropes?*”; 1 = *not important at all* to 7 = *very important*), and one item pitted the two motives against each other (“*In your imagination, was it more important to act precisely or to act collaboratively*?”; 1 = *precisely*, 7 = *collaboratively*). After reverse-coding the three accuracy-related items, the seven-item scores were averaged so that lower scores indicated an active accuracy motive, whereas higher scores indicated an active connectedness motive (Cronbach’s α = 0.80).

#### Persuasive Message and Dependent Variables

Participants read the same cover story and persuasive message, presented by the same groups, as in Study 1. Ratings of similarity to the source groups, of argument strength, as well as of the warmth and competence of the source groups were also the same as in Study 1. Changes were made to the assessment of attitudes and of thought favorability.

##### Product Attitudes

Five items were used to create a more reliable attitude index. In addition to rating the *likelihood of buying* and of *recommending* the product (as in Study 1), participants also rated *how useful* and *how interesting* the product was, as well as *how positive their overall impression* of the product was (response scales from 1 = *not at all*, to 7 = *very*). Again, excluding the “cost” item, the internal consistency of the attitude index was excellent, Cronbach’s α = 0.91.

##### Thought Favorability

The thought listing was changed insofar as participants were asked to go back to their listed thoughts and rate each thought on a scale from 1 = *very negative* to 7 = *very positive*. These ratings were averaged for each participant, across all thoughts listed, into a thought-favorability index. For two participants who had not listed any thoughts, the thought favorability index was considered neutral (=midpoint of the scale).

## Results and Discussion

### Effectiveness of Source Manipulation

In order to examine the effect of the source manipulation and whether motive priming changed the perception of the sources, we first conducted two 2 × 3 ANOVAs on warmth and competence, respectively, with motive priming (accuracy vs. connectedness) and sources (hc/lw, lc/hw, students) as between-subjects factors. Both the ANOVA on warmth, *F*(2, 197) = 42.60, *p* < 0.001, η^2^ = 0.302, 95% CI (0.197, 0.391), and on competence, *F*(2, 197) = 31.36, *p* < 0.001, η^2^ = 0.241, 95% CI (0.141, 0.332), returned significant effects for source perception. There was, however, no effect of motive-priming on perceived warmth or competence, both *F* < 1. In addition, there was no interaction of source and motive-priming regarding either perceived warmth, *F* < 1, or perceived competence, *F*(2, 197) = 1.26, *p* = 0.29.

In order to examine source perception on the level of the stereotyped groups, we conducted a series of *t*-tests. The lc/hw groups (housewives and disabled people) were once again perceived as higher in warmth (*M* = 5.24, *SD* = 0.88) than the hc/lw groups (career women and lawyers) (*M* = 3.90, *SD* = 1.03), *t*(200) = 9.24, *p* < 0.001, *d* = 1.45, 95% CI (1.11, 1.79). However, in contrast to Study 1, students (*M* = 4.75, *SD* = 0.77) were perceived as being less warm than the lc/hw groups, *t*(200) = −2.78, *p* = 0.006, *d* = −0.53, 95% CI (−0.91, −0.15). Nonetheless, they were still perceived as higher in warmth than the hc/lw groups, *t*(200) = 4.71, *p* < 0.001, *d* = 0.92, 95% CI (0.53, 1.31). As expected, the pattern was reversed for competence. The hc/lw groups (career women and lawyers) were perceived as being more competent (*M* = 5.38, *SD* = 0.95) than the lc/hw groups (housewives and disabled people) (*M* = 4.18, *SD* = 0.95), *t*(200) = 7.92, *p* < 0.001, *d* = 1.25, 95% CI (0.91, 1.58). Students (*M* = 4.73, *SD* = 1.01) were perceived as less competent than hc/lw groups, *t*(200) = −3.41, *p* = 0.001, *d* = −0.67, 95% CI (−0.28, −1.06), but as more competent than lc/hw groups, *t*(200) = 3.03, *p* = 0.002, *d* = 0.58, 95% CI (0.20, 0.95).

In terms of perceived similarity, participants reported higher perceived similarity to their ingroup (students; *M* = 4.65, *SD* = 1.61) relative to any of the other source groups (combined *M* = 2.93, *SD* = 1.44), *t*(198) = 6.55, *p* < 0.001, *d* = 2.32, 95% CI (1.58, 3.04).

### Effectiveness of Motive Priming

A 2 × 3 ANOVA on the index of accuracy vs. connectedness motivation with motive priming (accuracy vs. connectedness) and sources (hc/lw, lc/hw, students) as between-subjects factors showed that the motive priming was successful. Participants in the accuracy conditions (*M* = 3.25, *SD* = 1.27) scored significantly lower (=indicating an accuracy focus) than did participants in the connectedness conditions (*M* = 4.83, *SD* = 0.59), *F*(1, 197) = 109.13, *p* < 0.001, η^2^ = 0.356, 95% CI (0.254, 0.446). There were no further significant effects, both *F* < 1.

### Product Attitude

**FIGURE 1:**
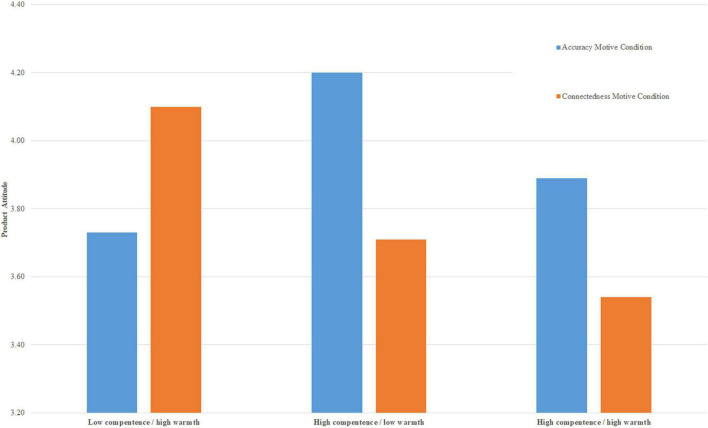
Condition means for product attitudes in Study 2. The lc/hw groups are comprised of housewives and disabled persons (averaged), the hc/lw groups are comprised of career women and lawyers (averaged); the hw/hc group is students.

First, we conducted an ANOVA on product attitude, including motive (accuracy vs. connectedness) and source groups (hc/lw, lc/hw^2^) as between-subject factors. The ANOVA returned no main effects for source, *p* = 0.87, or motive priming, *p* = 0.80, but a significant source by motive priming interaction, *F*(1, 159) = 4.04, *p* = 0.046, η^2^ = 0.025, 95% CI (0, 0.088). In order to achieve more fine-grained analyses of our hypothesis, we also computed a one-way ANOVA on product attitude across the six experimental conditions (made up by all possible combinations of the three levels of source: hc/lw, lc/hw, students, and the two levels of the motive condition: accuracy vs. connectedness), including an *a-priori* contrast that tested the hypothesized interaction pattern of lc/hw groups being most persuasive in the connectedness-motive condition and hc/lw groups being most persuasive in the accuracy-motive condition. The one-way ANOVA returned no significant omnibus effect, *F*(5, 197) = 1.16, *p* = 0.33. The contrast effect, however, was significant, *t*(197) = 2.02, *p* = 0.044, *d* = 0.64 95% CI (0.16, 1.26) (see Figure 1 for condition means), supporting our hypothesis. Further contrast tests showed that students were not more persuasive than either the hc/lw groups in the connectedness conditions or the lc/hw groups in the accuracy conditions, both *p* > 0.64.

### Thought Favorability

There was a significant correlation of thought favorability and product attitude, *r*(202) = 0.49, *p* < 0.001, 95% CI (0.38, 0.59). Different from product attitude, thought favorability was independent of source, motive, and the source × motive interaction. An ANOVA on the thought favorability index including motive (accuracy vs. connectedness) and source groups (hc/lw, lc/hw, students) as between-subjects factors returned no significant effects, all *p* > 0.11. We also conducted a one-way ANOVA across the six experimental conditions, in the same way as had been done for product attitude (see prev. paragraph), again including *a-priori* contrasts that tested the specific interaction pattern. However, the ANOVA returned no significant omnibus effect, *p* = 0.43, and no significant contrast effect, *p* = 0.75. When thought favorability was included as a covariate in a 2 × 2 ANCOVA on product attitude with motive (accuracy vs. connectedness) and sources (lc/hw vs. hc/lw^3^) as between-subjects factors, the results of the initial ANOVA replicated. Thought favorability was significant as a covariate, *F*(1, 158) = 53.93, *p* < 0.001, η^2^ = 0.254, 95% CI (0.145, 0.358), but its inclusion did not alter the pattern of effects. There were no main effects of either source or motive, both *p* > 0.35, but a significant interaction effect of both, *F*(1, 158) = 6.27, *p* = 0.013, η^2^ = 0.038, 95% CI (0.002, 0.111).

### Attitudes Toward Strong and Weak Aspects

We computed attitude indices toward aspects of the shower foam that were promoted with either only weak or only strong arguments. Then we conducted a 2 × 2 × 2 mixed-model ANOVA on attitudes toward strong vs. weak aspects as a within-subjects factor, using motive priming (accuracy vs. competence) and source (lc/hw vs. hc/lw^4^) as between-subjects factors. Aspects promoted with strong arguments were evaluated considerably more positively (*M* = 6.51, *SD* = 1.54) than aspects promoted with weak arguments (*M* = 4.24, *SD* = 1.71), *F*(1, 159) = 190.28, *p* < 0.001, η^2^ = 0.545, 95% CI (0.442, 0.621). In addition, averaged aspects of the product were evaluated more positively when the source had been a lc/hw group (*M* = 5.59, *SD* = 1.17) compared to a hc/lw group (*M* = 5.11, *SD* = 1.28), *F* (1, 159) = 6.19, *p* = 0.014, η^2^ = 0.037, 95% CI (0.001, 0.110). This effect, however, was considerably smaller than the effect of argument quality. There was also an interaction of argument quality and motive condition, *F*(1, 158) = 4.71, *p* = 0.031, η^2^ = 0.029, 95% CI (0.0, 0.097), indicating that participants in the accuracy conditions differentiated more between strong (*M* = 6.71, *SD* = 1.42) and weak arguments (*M* = 4.10, *SD* = 1.77) than did participants in the connectedness conditions (*M* = 6.30, *SD* = 1.63 vs. *M* = 4.39, *SD* = 1.64). This interaction, however, was independent of source condition, *F* < 1, for the three-way interaction; it may thus be taken as an additional indicator of the accuracy-motive manipulation having been effective.

### Source Group, Motive, and Thought Favorability as Concurrent Predictors of Product Attitude

To test if an interaction of source and motive conditions on product attitude would emerge even after controlling for thought favorability, we ran a hierarchical multiple regression analysis with product attitude as the dependent variable. In the first block, thought favorability, source condition (hc/lw vs. lc/hw; excluding the hc/hw condition), and motive condition (accuracy vs. connectedness) were entered as concurrent predictors. In the second block, a product term representing the motive-by-source interaction was entered as another predictor. The results are shown in Table 1. In the first block, only thought favorability emerged as a strong predictor of product attitude, whereas there were no main effects of either source or motive. In the second block, importantly, the motive-by-source interaction was predictive of product attitude even when the effect of thought favorability was controlled for. Thus, source effects appeared independent of systematic processing of the message.

**TABLE 1:** Multiple regression of product attitude on thought favorability, source condition, motive condition, and the source-by-motive interaction in study 2.

Block	Predictor	*R* ^2^	β	*95% CI*	*t*	*p*
Block 1		**0.244**				
	**Thought favorability**		**0.497**	**0.360, 0.634**	**7.165**	**<0.001**
	Source		0.068	−0.069, 0.205	0.976	0.331
	Motive		−0.005	−0.152, 0.122	−0.077	0.938
Block 2		**0.273**				
	**Source** × **Motive**		**0.549**	**0.115, 0.983**	**2.504**	**0.013**

*N = 162. Thought favorability ranges from 1 = very negative to 7 = very positive; source is coded 0 = low competence/high warmth, 1 = high competence/low warmth; motive is coded 0 = connectedness, 1 = accuracy. Significant effects are shown in boldface.*

### Perceived Warmth, Competence, and Thought Favorability as Concurrent Predictors of Product Attitude

As in Study 1, we also examined the role of perceived warmth and competence of the source in predicting product attitude. To do so, we conducted multiple regression analyses, separately for each motive condition, with thought favorability, perceived warmth, and perceived competence as predictors of product attitude. The results are shown in Table 2. In the accuracy-motive conditions, as in Study 1, the strongest predictor was thought favorability, followed by perceived competence; however, the effect of competence emerged only as a trend, and the negative effect of warmth we had observed in Study 1 also did not replicate. In the connectedness-motive conditions, the strongest predictor was again thought favorability, followed by a significant positive effect of perceived warmth; the effect of perceived competence was not significant. Thus, although the overall pattern was not very strong^5^, the relative contribution of perceived warmth versus competence in predicting product attitudes varied meaningfully with participants’ processing motive.

**TABLE 2:** Multiple Regression of Product Attitude on Thought Favorability, Perceived Warmth of Source, and Perceived Competence of Source, Reported Separately by Motive Condition, in Study 2.

Motive Condition	Predictor	β	*95% CI*	*t*	*p*
Accuracy	**Thought favorability**	**0.423**	**0.242, 0.604**	**4.676**	**<0.001**
	Warmth	0.130	−0.051, 0.311	1.426	0.157
	Competence	0.155	−0.026, 0.336	1.704	0.092
Connectedness	**Thought favorability**	**0.496**	**0.327, 0.665**	**5.831**	**<0.001**
	**Warmth**	**0.227**	**0.060, 0.394**	**2.712**	**0.008**
	Competence	0.079	−0.086, 0.270	0.926	0.357

*n = 101 for accuracy motive condition; n = 102 for connectedness motive condition. Thought favorability ranges from 1 = very negative to 7 = very positive; perceived warmth and competence both range from 1 = low to 7 = high. Significant effects are shown in boldface.*

### Discussion

We found plenty of evidence in support of our hypothesis that the relative influence of stereotyped groups would depend on recipients’ current motivation. Participants evaluated the shower foam more positively either when they were primed with an accuracy motive and influenced by a hc/lw source or when they were primed with a connectedness motive and influenced by a lc/hw source. There was, however, less support for the assumption of an ingroup being as influential as the ambivalent groups matching the activated motive. Descriptively, persuasion effects of a student source, presumed to represent a hc/hw group, appeared to be similar to those of the hc/lw groups. However, the ingroup was not significantly more influential than the ambivalent groups mismatching the activated motive. This underlines the finding of Study 1 that there are no additive effects of perceived warmth and competence of a source, but only the effects of the source dimension which matches the motivational context. In fact, the results even indicate that generally positively stereotyped groups may be less persuasive than ambivalent groups matching the activated motive. However, given the present data, we cannot determine whether this pattern may apply to positively stereotyped groups in general, or may be specific to the student (ingroup) source, in particular.

Furthermore, across processing motives, the source effects we observed were again *direct effects* on attitude judgments; different sources did not bias thought favorability, nor did they instigate more or less argument processing. As in Study 1, this pattern emerged in parallel to a pattern of systematic processing overall: Thought favorability was a function of argument strength, and was highly correlated with product attitude, independent of the effects of the warmth and competence dimensions. Independent of the message source, but in line with previous persuasion research, we found a marginal interaction of motive condition and message processing, which suggests more systematic processing when an accuracy motive was activated (cf. Petty et al., 1981).

## General Discussion

In two experiments, we found direct effects of sources stereotyped either as warm and incompetent or as competent and cold on the evaluation of a consumer product. In Study 1, where no specific motive had been primed, the competent but cold groups were more persuasive than the warm but incompetent groups. Furthermore, in Study 2, where accuracy vs. connectedness motives were primed, we found that the source effects depended on the participants’ current motive. If a connectedness motive had been primed, high warmth groups were more persuasive; if an accuracy motive had been primed, high competence groups were more persuasive. These effects were not mediated by thought favorability or amount of processing. Whereas in both studies there was evidence for a systematic processing of message arguments and for high correlations of message-related thought favorability and attitudes, these processes occurred in parallel and were independent of the main effect of the source (Study 1) and of the source-motive interaction effect (Study 2) on product attitudes.

We have discussed three potential ways in which source information can influence attitude judgments: in terms of direct main effects, *via* biasing thought favorability (Bohner et al., 1995), or by affecting the extent of processing (Baker and Petty, 1994). The present results suggest that the effect of ambivalently stereotyped groups primarily represents a direct influence of group characteristics as a source cue. However, we should exert caution regarding the generalizability of these effects. In other contexts, such as when a message contains ambiguous arguments (Chaiken and Maheswaran, 1994) or when baseline levels of processing are low (Maheswaran and Chaiken, 1991), effects of stereotyped group sources biasing or enhancing systematic processing might well be observed. This would be for future research to examine.

Returning to the present data, the absence of mediation by thought favorability and of interactions with argument quality suggests that neither biased nor increased (or decreased) processing was responsible for the source effects we observed. We have discussed the direct source effect reported in Study 1 as being incompatible with the assumption of a primacy of warmth (Cuddy et al., 2008), perhaps suggesting a primacy of competence or a context-sensitivity of source effects (i.e., with regard to the experimental setting; see Hilton and Darley, 1991; Bless et al., 2003). Indeed, when the results of Study 2 are taken into account, the present data support the assumption that, at least within a persuasion setting, there might be no universal primacy of either SCM dimension but instead a context-dependency of their effects (see also Bohner et al., 1995; Keller et al., 2000). The results also indicate that effects were not the result of concepts associated with the perception of competence, such as social status (Brinol and Petty, 2009).

How exactly the matching effect on participants’ attitudes was mediated is not entirely clear. One potential explanation would be that the effect was mediated by propositional thinking. Recipients under an accuracy motive may follow the heuristic that competent sources present accurate information, whereas recipients under a connectedness motive may follow heuristics, such as “I agree with people I like.” However, if this were the case, one might also expect that the favorability of participants’ thoughts would have been affected by the source manipulation, which was not the case. Given the absence of source effects on thought valence, a more plausible explanation might be that the matching effect on attitudes occurred at a less thoughtful and more associative level. The matching of motive condition and source might have increased the valence of the source, which was then attributed to the product, resulting in more positive evaluations. Future research might pinpoint the processing level at which attitude change occurs by including implicit measures alongside self-reports of attitude (Glaser et al., 2015; Linne et al., 2020).

Our finding that source effects can interact with recipients’ motives is not the first of its kind. For example, Imhoff and Erb (2009) showed that differences in recipients’ need for uniqueness predicted the relative persuasive impact of minority (vs. majority) sources. Taking a somewhat different approach, Dubois et al. (2016) showed that a matching (vs. mismatching) of power between sources and recipients led to greater persuasion; this matching effect was mediated by the use of competence-related (warmth-related) arguments by high-power (low-power) sources, along with the tendency of high-power (low-power) recipients to be more persuaded by competence-related (warmth-related) arguments. In contrast, our own studies conveyed the first evidence that broad source-related group stereotypes in terms of ascribed warmth and competence can, in meaningful interaction with the context (either an experimental situation or a matching motivation), influence the outcomes of persuasion by group sources. Indeed, our data showed that these fundamental dimensions of social judgment were even more influential than specific expertise regarding the attitude object. Given the general importance of these dimensions as representations of a group’s (or its members’) intentions and abilities (Fiske et al., 2002), this constitutes a major finding. As ambivalent combinations of source characteristics are rather the rule than an exception (Cuddy et al., 2008), it is particularly important to be able to align source with context characteristics in order to predict the success of persuasion attempts (Jones and Thibaut, 1958; Aaker and Lee, 2001; Bargh et al., 2001). Nevertheless, future research might also consider an in-depth analysis of groups stereotyped as either lc/lw or hc/hw. We have attempted to represent groups high on both dimensions by using an ingroup (Cuddy et al., 2008). Our results tend to support the notion of a specific persuasive appeal of either dimension, instead of additive effects, where the impact of high warmth and high competence would be combined into an even larger effect. Perhaps there is even a reduced effect of a source group positively stereotyped on both dimensions compared to ambivalently stereotyped source groups that are positively stereotyped on only one dimension. Becker and Asbrock (2012) have shown that behavior toward ambivalently stereotyped groups is mediated by the relative salience of warmth and competence. Similarly, persuasive influence might depend on dimension salience, favoring ambivalently stereotyped groups over generally positively stereotyped groups. Whereas, given that the context fits the dimension, ambivalently stereotyped groups may be characterized by their positively stereotyped dimension only, the effect of generally positively stereotyped groups may be impaired by a focus on a positive but situationally irrelevant second dimension of social judgment. Although our data hint at stronger persuasion by ambivalently stereotyped groups (vs. the student group), we have little evidence in favor of underlying processes driving the effect.

Although our data suggest stronger effects of competence than of expertise, more specific subdimensions (e.g., intelligence or likability; cf. Wallace et al., 2021) may come into play in certain settings. This may be the case, for example, when a group source is well-known, such as the student source in our current studies.

There are some limitations to the present studies, which should be addressed by future research. First and foremost, the statistical power of Study 1 was relatively low. We are aware of this limitation but believe that the effects found in our experiments are sufficiently strong to justify the conclusions we have drawn. Nonetheless, a successful replication using a larger sample would help to underline the validity of our findings. Furthermore, in the present experiments, we used the student source in a dual role: as an ingroup and as a hc/hw condition. Further research may instead systematically use all potential combinations of stereotyped groups (i.e., adding lw/lc groups) and use a source group that is attributed with high warmth and high competence not because of being an ingroup but, for example, because of the nature of their profession (e.g., physicians; Asbrock, 2010). This inclusion may be especially useful for further testing of specific vs. additive effects, as discussed above. In a similar vein, future research may aim to test the theoretical assumption with a broader stimulus range and a more diverse sample of participants. Other attitude objects than (only) a shower foam may be used as well as a greater quantity of source groups, including groups that are even more clearly stereotyped as warm or as competent. Finally, future research may address other types of matching effects involving stereotyped group sources. Whereas our research so far has addressed a matching of source and context, matching effects might also be found when group stereotypes about the source either match or mismatch attributes of the audience (Dubois et al., 2016) or the message content (for a review of related evidence, see Teeny et al., 2021).

To sum up, we were able to show that basic dimensions of social judgment (i.e., warmth and competence) do influence the processes of persuasion by group sources, and that which dimension is more influential depends on recipients’ current motivation. If recipients aim to make the correct decision, they will listen to group sources that are allegedly competent. If recipients strive for social connectedness, they will listen to group sources that are allegedly warm.

## Data Availability Statement

The data that support the findings of this research are available in PUB—Publications at Bielefeld University at https://pub.uni-bielefeld.de/record/2942938.

## Ethics Statement

The studies involving human participants were reviewed and approved by Ethics Committee of the Faculty for Psychology and Sports Science, Bielefeld University. Written informed consent from the participants’ legal guardian/next of kin was not required to participate in this study in accordance with the national legislation and the institutional requirements.

## Author Contributions

RL, MS, and GB contributed to the manuscript. MS conducted the original research and designed the experiments and collected the data. RL performed the statistical analyses (with support from GB) and wrote the first draft of the manuscript. RL and GB contributed to the manuscript revision. All authors approved the submitted version.

## Conflict of Interest

The authors declare that the research was conducted in the absence of any commercial or financial relationships that could be construed as a potential conflict of interest.

## Publisher’s Note

All claims expressed in this article are solely those of the authors and do not necessarily represent those of their affiliated organizations, or those of the publisher, the editors and the reviewers. Any product that may be evaluated in this article, or claim that may be made by its manufacturer, is not guaranteed or endorsed by the publisher.

## Footnotes

1 We chose these items instead of more standard attitude items (e.g., “Do you like…?”) because of their fit with the presented cover story. Although, in a narrow sense, they are behavioral expectation items, we believe them to be indicative of participants’ attitudes.

2 When students are included in the ANOVA, the interaction is no longer significant, *p* = 0.10.

3 The interaction remains significant when students are included in the ANOVA, *F*(2, 196) = 3.44, *p* = 0.034, η^2^ = 0.034, 95% CI (0, 0.091).

4 When students are included in the ANOVA, only the main effect for argument strength, *p* < 0.001, and the main effect for source, *p* = 0.029 (students, *M* = 5.21, *SD* = 1.50), remain significant.

5 Indeed, a full regression model did not yield significant interaction effects of either perceived warmth or perceived competence with the motive condition, *F*(17, 123) = 1.48, *p* = 0.114, η^2^ = 0.170, and *F*(17, 123) = 0.98, *p* = 0.468, η^2^ = 0.121, respectively.
