# Supplementary material for: Ambivalent Stereotypes and Persuasion: Attitudinal Effects of Warmth vs. Competence Ascribed to Message Sources
Source: Front Psychol. 2022 Jan 27;12:782480. doi: 10.3389/fpsyg.2021.782480 (PMC8830407; doi:10.3389/fpsyg.2021.782480)
Supplement: Supplementary file 1 [file Data_Sheet_1.pdf]

Online Supplement

Pilot Studies

To generate the materials for Experiments 1 and 2, three pilot studies were conducted with samples from the same student population.

Pilot Study 1: Social Groups

The first pilot study was designed to select, as sources of persuasion, social groups that represent the ambivalent clusters of the SCM. Additionally, we tested the suitability of the group *students* for use as a hc/hw source. In an online questionnaire, 60 students from Bielefeld University (24 women, 36 men; mean age 24.03 years, *SD* = 6.33, range 17 to 60) rated 13 social groups regarding their perceived warmth (items: *likable*, *warm-hearted*, *good-natured*, and *friendly*) and competence (items: *competitive*, *competent*, *independent*, and *intelligent*). The response scale went from 1 = *not at all*, to 7 = *completely*. Responses were averaged across items to form indices of warmth and competence, respectively, for each target group. Based on the results, five groups were retained for use in Studies 1 and 2 (see Online Supplement): Two groups (*housewives* and *disabled people*) were chosen as prototypical examples of lc/hw groups, and two groups (*career women* and *lawyers*) were chosen as prototypical examples of hc/lw groups. As expected, the group of *students* was rated high on both dimensions (hc/hw).

Pilot Study 2: Arguments

We generated thirteen arguments that could form part of a recommendation of the shower foam. Each addressed a specific aspect (e.g., antiperspirant effect; eco-friendliness) and was initially written in a stronger (e.g., “95% of the shower foam is biodegradable”) and a weaker version (e.g., “a small amount of the shower foam is biodegradable”). One of two online questionnaires that each contained roughly equal numbers of stronger and weaker argument versions, but never the stronger and weaker version of the same argument, was completed by 55

participants (19 women, 36 men; mean age 23.87 years, *SD* = 5.06, range 17 to 47; *n* = 31 and 24, respectively). They rated each argument on a scale from 1 = *not at all convincing* to 9 = *very convincing*) In addition, they reported their agreement with each of the specific aspects that the arguments represented (e.g., “this shower foam is eco-friendly”; 1 = *completely disagree* to 9 = *completely agree*). We selected strong, moderate, and weak arguments based on one-sample *t*-tests that compared both the argument ratings and the aspect ratings with their scale midpoint of 5. This yielded three moderate arguments whose ratings were close to the midpoint of both scales, two strong arguments whose ratings were significantly higher than the midpoint of both scales, and two weak arguments whose ratings were significantly lower than the midpoint of both scales (see Online Supplement).

Pilot Study 3: Specific Expertise and Identification

In a final pilot study, we assessed ratings of the source groups' specific expertise regarding the shower foam, and participants' identification with those groups. Participants (*N* = 32, 17 women, 15 men; mean age 22.78 years, *SD* = 3.82, range 17 to 33) were presented with several products (e.g., coffee, LED-flashlights), including the target product "shower foam". For each product, they rated how *expert* each of twelve groups, including the five source groups selected from Pilot Study 1, is with respect to the specific product (scale from 1 = *not at all* to 7 = *entirely*). Results showed that the highest product-specific expertise was ascribed to housewives, followed by career women, students, lawyers, and disabled people (see below, pilot ratings of expertise and identification). The means of product-specific expertise for the five source groups thus were not aligned with either general competence or warmth. Participants also rated how much they identified with each of the groups (scale from 1 = *not at all* to 7 = *entirely*). Results revealed that participants identified strongly with *students* (*M* = 6.09) but did not identify much with any of the other groups (all *M* # 3.03; see below, pilot ratings of expertise and identification).

Pilot Ratings of Warmth *Pilot Ratings of Warmth (4-Item Index) and Competence (4-Item Index) of Five Influence Groups (Pilot Study 1)*

	Warmth		Competence	
Source group	*M* (*SD*)		*M* (*SD*)	
Housewives	5.33_{a}	(0.95)	3.78_{c}	(0.95)
Disabled people	$5.06_{a,b}$	(1.00)	2.51_{d}	(0.90)
Career women	3.46_{c}	(1.20)	5.77_{a}	(1.03)
Lawyers	3.06_{c}	(1.14)	5.73_{a}	(0.98)
Students	4.68_{b}	(0.92)	4.90_{b}	(0.94)

Note. $N = 60$. All ratings were made on scales from 1 to 7. The warmth index consists of the items *likable*, *warm-hearted*, *good-natured*, and *friendly*; the competence index consists of the items *competitive*, *competent*, *independent*, and *intelligent*. Cronbach's alpha of the warmth and competence indices ranged from .60 (housewives-competence) to .90 (lawyers-warmth). Within a given column, means not sharing a subscript differ at $p < .05$, Bonferroni-adjusted pairwise comparisons based on paired-sample *t*-tests.

Pilot Ratings of Shower Foam Arguments *Pilot Ratings of Shower Foam Arguments That Were Selected and of Associated Aspects (Pilot Study 2)*

Argument strength	Content (aspect)	Argument rating *M (SD)*	Aspect rating *M (SD)*
Moderate	Integrated deodorant prevents sweating for 48 hrs (antiperspirant effect)[a]	4.65 (2.69)	5.45 (2.17)
	May be used as shampoo to give hair a healthy shine (hair)[a]	5.48 (2.42)	5.52 (2.23)
	Partly biodegradable, thus protects the environment (eco-friendliness)[b]	5.17 (2.55)	4.42 (2.26)
Strong	Excellent cleansing effect, skin feels thoroughly clean after showering (cleansing effect)[a]	6.61** (1.89)	6.61** (2.06)
	Nutritional effect renders skin tender and soft (care)[b]	7.21** (1.84)	6.71** (1.57)
Weak	Just a few Euros more expensive than a regular shower foam (price)[a]	2.35** (1.96)	2.19** (1.40)
	Container must be shaken for one minute to produce foam (handling)[b]	1.83** (1.37)	3.75* (2.05)

Note. All ratings were made on scales from 1 to 9.

[a]Data based on n = 31.

[b]Data based on n = 24.

* Difference from the scale midpoint of 5 is significant at $p < .01$.

** Difference from the scale midpoint of 5 is significant at $p < .001$.

Pilot Ratings of Expertise and Identification *Pilot Ratings of Product-specific Expertise of and Identification With Five Influence Groups (Pilot Study 3)*

	Product-specific expertise		Identification	
Source group	*M* (*SD*)		*M* (*SD*)	
Housewives	5.41_{a}	(1.21)	$2.41_{b,c}$	(1.74)
Disabled people	3.72_{c}	(1.55)	2.00_{c}	(1.61)
Career women	$5.06_{a,b}$	(1.41)	3.03_{b}	(1.89)
Lawyers	$4.75_{a,b}$	(1.41)	$2.87_{b,c}$	(2.23)
Students	4.78_{b}	(1.41)	6.09_{a}	(1.47)

Note. $N = 32$. All ratings were made on scales from 1 to 7. Within a given column, means not sharing a subscript differ at $p < .05$, Bonferroni-adjusted pairwise comparisons based on paired-sample *t*-tests.

Ratings of Warmth and Competence in the Present Studies

Ratings of Warmth and Competence of All Influence Groups in Study 1

	Warmth		Competence	
Source group	*M* (*SD*)		*M* (*SD*)	
Housewives	5.15	(1.08)	4.57	(1.34)
Disabled people	4.91	(1.00)	3.86	(1.16)
Career women	3.79	(0.97)	5.71	(0.86)
Lawyers	3.54	(0.65)	5.51	(1.00)
Students	5.23	(0.83)	5.03	(1.19)

Note. $N = 92$.

Ratings of Warmth and Competence of All Influence Groups in Study 2

	Warmth		Competence	
Source group	*M* (*SD*)		*M* (*SD*)	
Housewives	5.28	(0.86)	4.37	(1.05)
Disabled people	5.19	(0.91)	3.96	(0.78)
Career women	4.18	(0.93)	5.53	(1.04)
Lawyers	3.70	(1.06)	5.26	(0.87)
Students	4.75	(0.77)	4.73	(1.01)

Note. $N = 203$.
